# Links between observational measures of children’s emotion and reactive versus proactive aggression

**DOI:** 10.1017/S0954579426101394

**Published:** 2026-03-30

**Authors:** Julie A. Hubbard, Christina C. Moore, Lindsay Zajac, Megan K. Bookhout, Mary Dozier

**Affiliations:** 1 Psychological and Brain Sciences, University of Delaware, USA; 2 Dartmouth Hitchcock Medical Center, USA; 3 ABC Parenting Institute, USA; 4 The University of North Carolina at Chapel Hill, USA

**Keywords:** emotion, observational, proactive aggression, reactive aggression

## Abstract

Theorists conceptualize reactive aggression as emotional (especially angry) and proactive aggression as unemotional (although it is unclear whether relations between proactive aggression and emotion are null or negative). Goals of the current study were to: (a) examine links between reactive aggression and a range of emotions (happiness, sadness, anger, and anxiety), and (b) include neutral emotion to address whether proactive aggression is unrelated or negatively related to emotion. To assess emotion, playgroups of four same-sex, unfamiliar, nine-year-old children (N = 158; 52.5% males; racially/ethnically diverse) interacted as round-robin dyads while completing challenging and cooperative tasks; observers coded emotions second-by-second. To assess both behavioral and observational reactive-versus-proactive aggression, children completed video games with virtual peers. Reactive aggression was positively related to happiness, anger, and anxiety and negatively related to neutral emotion, for at least one task and one aggression measure. Proactive aggression was positively related to neutral emotion but negatively related to happiness, for both tasks and aggression measures. Findings enhance theoretical understanding of: (a) reactive aggression as broadly emotional by relating it to happiness and anxiety as well as anger, and (b) proactive aggression as unemotional by linking it to the display of neutral emotion and the lack of display of happiness.

Emotion is critical to the distinction between reactive and proactive aggression in youth. Theoretical descriptions of reactive aggression as emotional are backed by substantial evidence, especially for anger. Similarly, strong support relates proactive aggression to lack of emotion, although it is unclear whether the two constructs are unrelated or negatively related. However, most investigations assess both emotion and the functions of aggression via questionnaires, with only a few studies employing observational or behavioral measures of either construct. For these reasons, the three goals of the current study were to: (a) investigate the link between children’s emotion and the functions of aggression using observational or behavioral measures of both constructs, (b) examine associations between a range of emotions beyond anger and reactive versus proactive aggression, and (c) address whether proactive aggression is unrelated or negatively related to emotion through the measurement of neutral emotion.

## Reactive aggression and heightened emotion

Reactive aggression is retaliatory behavior in response to provocation (Dodge, 1991); the construct is based in the frustration-aggression hypothesis (Berkowitz, 1993). Reactive aggression is most closely tied to the emotion of anger, both theoretically and empirically. This link between anger and reactive aggression, but not proactive aggression, emerges across developmental periods (preschool: e.g., Song et al., 2020; middle childhood: e.g., McAuliffe et al., 2006; adolescence: e.g., Marsee & Frick, 2007), in both cross-sectional (e.g., Jambon et al., 2019) and longitudinal work (e.g., Ostrov et al., 2013), and cross-culturally (Spain: Calvete & Orue, 2012; Hong Kong: Fung et al., 2015; China: Xu et al., 2009). A recent daily diary study highlighted the comprehensive nature of the association, with reactive aggression predicting higher levels of daily anger, more variability in anger across days, and heightened angry reactivity to negative events (Moore et al., 2019).

One explanation for the connection between anger and reactive aggression focuses on hostile attributional biases, a construct closely linked to reactive aggression, but not proactive aggression (see Orobio de Castro & van Dijk, 2018 for a review). Hostile attributional biases and anger co-occur in real time, with the linkage predicting reactive but not proactive aggression (Yaros et al., 2014). In addition, intervention efforts to decrease these biases also effectively reduce anger and reactive aggression in youth (see Powell et al., 2011 for a review).

However, reactive aggression may be tied not only to anger, but to heightened negative emotion more broadly (Berkowitz & Harmon-Jones, 2004). Empirically, reactive but not proactive aggression in youth has been associated with depressive symptoms (e.g., Preddy et al., 2014; Yang et al., 2024), anxious symptoms (e.g., Fite et al., 2014; Fung et al., 2015), suicidality (e.g., Hartley et al., 2018), and increased variability in daily fear (Moore et al., 2019). The association between reactive aggression and negative emotion may include not only elevated levels of negative affect, but increased negative emotional lability, as seen in a recent study using ecological momentary assessment (Slaughter et al., 2020). Finally, the connection between reactive aggression and emotion may extend to positive emotions. In the diary study described above, although reactive aggression was related to lower overall levels of daily happiness, it also was linked to greater happy reactivity when positive events occurred (Moore et al., 2019).

The relation between reactive aggression and heightened emotion may be explained in terms of both regulatory and physiological processes. Investigations have linked reactive aggression to deficits in emotion regulation (e.g., Marsee & Frick, 2007; Ostrov et al., 2013) and effortful control (e.g., Dane & Marini, 2014; Rathert et al., 2011), with one study suggesting that the longitudinal association between anger and reactive aggression is stronger for youth with poorer emotion regulation skills (Calvete & Orue, 2012). These deficits may be driven both by decreased functioning of the parasympathetic nervous system and hyperarousal of the sympathetic nervous system, two physiological processes related to reactive aggression specifically (Hubbard et al., 2002; Moore et al., 2018; Scarpa et al., 2010; Xu et al., 2014).

## Proactive aggression and lack of emotion

In contrast to reactive aggression, youth proactively aggress deliberately to reach an instrumental or social goal (Dodge, 1991). Proactive aggression is theoretically based in social learning theory (Bandura, 1973), and this learned behavior is linked to both positive outcome expectations for aggressive behavior (Orobio de Castro & van Dijk, 2018) and prioritizing instrumental over social goals (e.g., Salmivalli et al., 2005). Moreover, proactive aggression is associated with problems identifying peers’ sad or fearful facial expressions (Dadds et al., 2006; Marsh & Blair, 2008) and difficulties experiencing sympathy for victims (Jambon et al., 2019), deficits which may make the negative social consequences of aggression less compelling.

This description of calculated aggression for gain suggests that children and adolescents who enact proactive aggression are able to remain calm and composed while doing so, and the null findings between proactive aggression and emotion described in the previous section back up this assertion. Explanations for the link between proactive aggression and unemotionality include links between this function of aggression and both strong emotion regulation skills (Ostrov et al., 2013; Rathert et al., 2011) and low baseline autonomic functioning (e.g., Raine et al., 2014; Scarpa et al., 2010). Perhaps most persuasive is a recent study relating proactive aggression to both low levels of sympathetic arousal and high levels of parasympathetic functioning in the moment that the aggressive behavior occurred (Moore et al., 2018).

Beyond social learning theory, scholars’ theoretical framework for proactive aggression emphasizes both fearlessness and sensation seeking. Youth who engage in proactive aggression may experience low levels of fear, minimizing the negative outcomes of aggression and reinforcing the positive outcomes, resulting in socialization deficits (Fung et al., 2005; Raine, 2002). In fact, connections between fearlessness and proactive but not reactive aggression are supported empirically (Kimonis et al., 2006). Moreover, if youth who engage in proactive aggression experience lower baseline physiological arousal than their peers, they may be motivated to seek out stimulating experiences, including aggression, to increase their arousal (Raine, 2002). Indeed, empirical associations between sensation seeking and proactive aggression in particular have been reported (Xu et al., 2014), with especially compelling studies suggesting that sensation seeking mediates the link between low resting heart rate and proactive aggression both concurrently (Portnoy et al., 2014) and longitudinally (Sijtsema et al., 2010).

Of note, the literature on proactive aggression and lack of emotion, as well as the literature on reactive aggression and heightened emotion reviewed in the previous section, encompasses both studies that take a trait approach and studies that take a state approach to the measurement of emotion. A detailed discussion of this distinction is beyond the scope of the current paper, because addressing it adequately would require an analysis of design and timescale used in the assessment of emotion in each study. However, it is important to note that these findings emerge across both approaches to the assessment of emotion.

## Observational/behavioral assessment of emotion and the functions of aggression

As described above, a substantial literature supports the emotional nature of reactive aggression and the unemotional nature of proactive aggression. However, most studies relied on questionnaire assessments of both constructs from a single reporter, usually the self (e.g., Fite et al., 2014; Marsee & Frick, 2007; Yang et al., 2024). Other investigations used different reporters for each construct (e.g., Jambon et al., 2019; Preddy et al., 2014; Vitaro et al., 2002), with one such study assessing self-reported emotion through daily diary methodology (Moore et al., 2019) and another measuring parent-reported emotion via ecological momentary assessment (Slaughter et al., 2020). Still other investigations gathered data from multiple reporters for each construct (McAuliffe et al., 2006; Xu et al., 2009). However, the fact remains that most studies of emotion and the functions of aggression have been conducted using questionnaire measures.

Additional evidence comes from studies linking psychophysiology, as an index of emotion, to the functions of aggression. In these investigations, the functions of aggression have been assessed via self report (Kruter et al., 2023), teacher report (Hubbard et al., 2002; Xu et al., 2014), or parent report (Raine et al., 2014; Scarpa et al., 2010; Song et al., 2020) while physiological constructs were measured either at baseline or in response to stressors.

To our knowledge, only three studies of emotion and both functions of aggression in youth have taken an observational or behavioral approach to the measurement of either construct. In a four-month longitudinal study, Ostrov and colleagues (2013) demonstrated that observations of preschoolers’ reactive aggression predicted increases over time in teachers’ ratings of anger and emotion dysregulation, whereas observations of proactive aggression predicted decreases in these variables. Hubbard and colleagues (2002) established that, the higher teachers’ reports of children’s reactive but not proactive aggression, the more anger they were observed to express over the course of a game as they were provoked by a peer. Finally, Moore and colleagues (2018) assessed children’s physiology at the same moment in time as they assessed their reactive and proactive aggression both observationally (via coding of verbal aggression) and behaviorally (through measurement of damage to a virtual peer’s drawing). Their findings suggested that skin conductance negatively predicted in-the-moment proactive aggression, whereas RSA moderated the in-the-moment relation between skin conductance and reactive aggression, such that the association was positive at low levels of RSA but negative at high levels of RSA.

The first goal of the current study was to investigate connections between children’s emotion and reactive versus proactive aggression using observational or behavioral assessments of both constructs. Although these links are well-established through other methods, given their theoretical importance, our confidence in these findings would be enhanced through further investigation of both emotion and aggression using observational or behavioral methods.

## Reactive aggression and a range of emotions

As described above, reactive aggression may be linked not only to anger, but to heightened emotion more broadly. To understand the range of emotions tied to reactive aggression, we must assess discrete emotions within the same study. To our knowledge, the only prior study to take this approach was the daily diary study by Moore and colleagues (2019), in which youth rated their happiness, sadness, anger, and fear on a daily basis. The second goal of the current study was to apply a discrete emotions approach (Izard, 1993) to observations of children’s happiness, sadness, anger, and anxiety. By linking these observed emotion variables to children’s reactive aggression, we hoped to enhance our understanding of the role that a range of emotions play in reactive aggression. Moreover, the use of situational measures of emotion allowed us to tap into the construct of emotional reactivity, since children were responding to incidents or interactions that unfolded in the dyadic tasks to be described below. This approach seemed especially useful, given that Moore and colleagues’ (2019) daily diary study revealed that reactive aggression was linked to greater happy reactivity when positive events occurred, even though it was also related to lower overall levels of daily happiness.

## Proactive aggression and neutral emotion

When scholars describe proactive aggression as unemotional, two possible interpretations fit this description, with the first suggesting a null relation between proactive aggression and emotion and the second suggesting a negative relation. Both interpretations are supported empirically, with studies suggesting that proactive aggression is unrelated to emotion or psychophysiology in some cases (e.g., Calvete & Orue, 2012; Fite et al., 2014; Hubbard et al., 2002; Jambon et al., 2019; Slaughter et al., 2020; Song et al., 2020; Vitaro et al., 2002; Xu et al., 2009, 2014), but negatively related in other cases (e.g., Ostrov et al., 2013; Preddy et al., 2014).

To address these inconsistent findings, we included an assessment of children’s neutral emotion (when they are not expressing either positive or negative emotion) in our observational coding, an approach which our literature review suggests is novel. Leading emotion theorists argue that individuals experience neutral emotion, the neutral valence of this affective state provides important information, and this information influences both cognition and behavior, positions they support with empirical evidence (see Gasper et al., 2019 for a review). Using this strategy, our final goal was to advance our understanding of whether children’s proactive aggression is negatively associated with positive and/or negative emotion, unrelated to positive and/or negative emotion, positively associated with the display of neutral emotion, or some combination of these possibilities.

## The current study

To summarize, the three goals of the current study were to: (a) investigate connections between children’s emotion and the functions of aggression using observational or behavioral assessments of both constructs, (b) include the observation of happiness, sadness, anger, and anxiety to advance our understanding of the range of emotions linked to reactive aggression, and (c) incorporate the observation of neutral emotion to address the question of whether proactive aggression is unrelated or negatively related to emotion. We hypothesized that: (a) reactive aggression would be positively related and proactive aggression would be negatively related to happiness, sadness, anger, and anxiety, and (b) proactive aggression would be positively related and reactive aggression would be negatively related to neutral emotion.

## Method

### Overview

Children participated in a broader longitudinal study described in more detail below that included laboratory visits at ages 9 and 10. At age 9, we observationally coded children’s emotion with peers during dyadic interactions. At age 10, we measured children’s reactive and proactive aggression, in terms of both behaviors and verbalizations, using standardized computer games. Only children who participated in both procedures are included in analyses reported here.

### Participants

Participants included 158 children (52.5% male; sex from parent report) from urban and suburban areas of a mid-Atlantic state in the United States. Parents reported children’s race as 60% African American, 18% Mixed race, 15% European American, and 7% Other and children’s ethnicity as 23% Latino/a and 77% Not Latino/a. Children’s age averaged 9.51 years (*SD*= .36) when we assessed emotion and 10.61 years (*SD*= .37) when we assessed aggression.

Families reported annual income averaging $40,616 (*SD* = $41,112), and 46% of families received welfare benefits. In terms of educational level, 22% of reporting parents did not complete high school, 10% earned a GED, 32% graduated from high school, 21% attended some college, 10% graduated from a four-year college, and 5% held a post-graduate degree.

Most participants were enrolled in a larger longitudinal study on the efficacy of a parenting intervention ((Attachment and Biobehavioral Catch-Up (ABC); Dozier & Bernard, 2019)) on middle childhood outcomes. We designed procedures both to assess intervention effects on peer outcomes and to address the question posed in this paper. Ninety-six children were recruited as infants through referrals from Child Protective Services (CPS) due to allegations of maltreatment. They were randomized to receive either ABC (*N* = 45) or control intervention Developmental Education for Families (DEF; *N* = 51). ABC is a 10-session home-based intervention designed to increase nurturance when children are distressed, increase sensitivity to children’s cues, and decrease frightening and harsh behaviors. DEF followed the same format but focused on teaching parents about child development. Children were followed until middle childhood, when a comparison sample (*N* = 62) was recruited through community centers and schools and matched to the intervention sample on race/ethnicity and sex.

CPS-referred and comparison children did not differ on any of the emotion or aggression variables described below (see Supplemental Materials A). In addition, the children whose parents completed ABC did not differ from those whose parents completed DEF on study variables (see Supplemental Materials B). For these reasons, we analyzed the entire sample.

### Procedures and measures

We obtained parental consent and child assent in writing for all procedures. The Institutional Review Board of the University of Delaware approved all procedures (Approval number 547621-18).

#### Emotion

*Dyadic interaction procedures.* Participants in these procedures included the 158 children described above, along with 44 additional nine-year-old children. In the summers of 2015–2017, children came to the lab in groups of four, and they were unfamiliar to one another. Given that most peer interactions during this developmental period occur within sex, groups were same-sex (Rose & Smith, 2018). Children provided verbal confirmation that they did not know each other at the beginning of each dyadic interaction described below.

All interactions occurred in dyads, and dyads did not interact prior to or between recorded interactions. Each child interacted with three partners in a round-robin manner (six dyads per group). First, Children A and B interacted in one room, while Children C and D interacted in a separate room. Children then switched partners, so that Children A and C could interact at the same time as Children B and D. Finally, children switched partners once again to give Children A and D an opportunity to interact, as well as Children B and C.

Each dyad completed two 5-minute tasks designed to elicit a range of emotions. In the first task, children searched for a missing object. Dyads AB and CD attempted to unlock a box by searching for a key that fit on a ring of hundreds of keys; however, the correct key was not included. Dyads AC and BD searched for a squirrel in a book with hundreds of animal photos, but no squirrel. Dyads AD and BC tried to find a ball with a smiley face in a bin of hundreds of balls, all without a smiley face. In the second task, children planned the perfect event (the perfect party for Dyads AB and CD, the perfect school for Dyads AC and BD, and the perfect field trip for Dyads AD and BC).

We did not counterbalance tasks across dyads or groups, given that tasks were analogous and dyads were interchangeable. Due to changes early in data collection, the first two groups completed only the search tasks and did not complete the planning tasks.

Before the tasks, children learned that they would earn tickets based on their performance and a prize if they accumulated 30 tickets. Before each search task, the experimenter told children that they would earn 10 tickets each if they found the missing object, but of course, no dyad earned these tickets. Before each planning task, the experimenter stated that children would earn tickets based on the quality of their ideas. In truth, each dyad’s ticket total for each planning task was predetermined so that each child accumulated 30 tickets and earned a prize.

A total of 52 playgroups participated, and 46 groups including four members. Scheduling difficulties resulted in six groups of only three children (for a total *N* of 202). For these six groups, each child interacted with two partners, while the other child simply waited. Ticket totals for planning tasks were modified so that children still earned prizes.

*Observational coding and measurement of emotion.* All interactions were videorecorded without children’s awareness and coded using Noldus The Observer XT Version 11. Observers coded one child at a time, with different observers coding each dyad partner. Coding included five emotions (Happiness, Sadness, Anger, Anxiety, Neutral). Observers identified each child’s primary emotion for each second of each task using the D.O.T.S. Emotion Coding System (Cole et al., 2007). Observers chose the emotion that best represented the child at that second based on facial expression, tone of voice, and body language. When children displayed blended emotions, coders selected the primary emotion.

A graduate student served as the trainer and gold standard for undergraduate observers, who were naïve to study hypotheses. During training, we compared observers’ and the trainer’s coding of pilot videos. Coders were deemed reliable against the trainer when they reached an overall Cohen’s kappa of at least .75 across emotions and an individual Cohen’s kappa of at least .75 for each emotion for five consecutive video segments (one dyad member completing one task). Once observers started to work independently, we made regular random reliability checks to assess for drift, and when we observed drift, we retrained. For the complete data set, 20% of interactions were coded by two observers who did not know which segments acted as reliability trials. Final Cohen’s kappa was .83 across emotions, .80 for Happiness, .99 for Sadness, .95 for Anger, .96 for Anxiety, and .75 for Neutral Emotion.

Emotion data for each child for each 5-minute task were aggregated into ten 30-second intervals. Variables (Happiness, Sadness, Anger, Anxiety, Neutral Emotion) were represented as the percentage of the interval that the child expressed that emotion. Given the brief timeframe of this measurement, it is best considered a measure of state emotion as opposed to trait emotion.

#### Proactive and reactive aggression

*Video game procedures.* The 158 children described in the *Participants* section completed two five-minute video games. Before each game, an experimenter introduced the participant to a different same-age, same-sex peer; although participants believed that these peers were real children taking part in the study at another university, in truth, they were simulated. The experimenter explained the rules described below and then left the participant alone to complete the game. In each game, the participant and virtual peer played as astronauts who earned points by pressing the left or right button on an Xbox controller as soon as they determined the direction of a star. In addition, both the participant and virtual peer had use of a foot pedal to “zap” the other’s astronaut, making the other child unable to earn points as long as the pedal was pressed. If participants pressed their pedal, the virtual peer responded with comments such as “Don’t do that!”, “Why are you being so mean?”, and “Hey, that’s not fair!” to ensure that participants understood that the virtual peer viewed pedal pressing as aggressive.

Although the two games were alike in all ways described above, they differed in several important aspects, such that the first game assessed proactive aggression whereas the second game assessed reactive aggression. In the proactive aggression game, the participant played *against* the virtual peer to determine who could earn the most points and win a chosen prize. Scores for both players were displayed on the screen. In addition, the virtual peer never used his/her foot pedal to prevent the participant from earning points. The game was fixed so that the participant always earned more points and won the prize.

In the reactive aggression game, the participant and virtual peer played *separate* games simultaneously. Each child could win money based on how many stars he/she collected, with each star worth ten cents. The participant’s screen displayed his/her total earnings thus far; however, the virtual peer’s total was not displayed, since the children were not in competition. However, both children still had use of the foot pedal to prevent the other child from earning money. In this game, the virtual peer pressed his/her foot pedal ten times. This game was also fixed, in that participants always earned $4 at the end of the game.

Of note, in the first game, the participant did not experience peer provocation (the virtual peer never prevented the participant from earning points), but he/she had an instrumental incentive to aggress (preventing the virtual peer from earning points increased the chance that the participant would win the game and prize). In contrast, the second game involved peer provocation (the virtual peer pressed his/her pedal ten times) but no instrumental gain from aggression (because the children were not competing against one another). Thus, the two games were designed such that, if participants aggressed, their aggression could be clearly labeled as proactive or reactive in function depending upon the game in which it occurred.

Although it would have been desirable to counterbalance game order, we ultimately decided not to do so. We reasoned that, if children first played with a provocative virtual peer, they might anticipate provocation from the second virtual peer, making it more difficult to disentangle the functions of aggression in each game.

Parents were fully informed of these procedures during the consent process. Children also signed an assent form explaining the voluntary nature of their participation, the ability to stop participation at any time, and confidentiality. However, participants were not told about the virtual peers or provocation beforehand. At the conclusion of the laboratory visit, participants were fully debriefed using the following language: “In the games, it seemed like you were playing with another kid, but really you were just playing with the computer. We wanted you to think the other player was real so that we could learn more about how children play with each other, especially when the other player acts in a way that isn’t nice. We do this with all the kids your age who come visit us, and kids are often surprised to find out that it was actually just a computer. Does that make sense to you? Do you have any questions?”

*Measurement of behavioral proactive and reactive aggression.* For the first game, a variable termed Behavioral Proactive Aggression was created representing the number of seconds (out of a possible 300) that the participant pressed the foot pedal. For the second game, an analogous variable was created termed Behavioral Reactive Aggression.

*Observational coding and measurement of verbal proactive and reactive aggression.* We videorecorded participants as they played both games for coding of their verbalizations using Noldus The Observer XT Version 11. Observers transcribed participants’ remarks verbatim and segmented them into “blocks,” with a new block beginning after pauses of at least two seconds. Observers then coded each block as Verbal Proactive Aggression, Verbal Reactive Aggression, or Verbal Other. For a block to be coded as Verbal Proactive Aggression, it needed to include both aggressive content (statements intended to threaten, hurt, intimidate, antagonize, mock, or insult the virtual peer) and an articulation of a desire to gain points or win the game (“I’m zapping you because I want to win!”). For a block to be coded as Verbal Reactive Aggression, it needed to include both aggressive content and an articulation of a desire for retaliation (e.g., “You do it to me, I’m gonna do it back”). We decided to require an explicit articulation of reactive or proactive intent along with aggressive content to ensure rigorous measurement of verbal aggression that was reactive or proactive in its function. If the content changed mid-block, observers parsed the verbalization into multiple blocks and assigned a unique code to each block. Of note, we did not analyze the Verbal Other category but included it to create an exclusive and exhaustive coding scheme.

A graduate student served as the trainer and gold standard for undergraduate observers, who were naïve to study hypotheses. Using pilot videos, we considered coders reliable against the trainer when they achieved an overall Cohen’s kappa of at least .75 across verbalizations and an individual Cohen’s kappa of at least .75 for each verbalization for five consecutive video segments (one participant completing one game). Once coders started independent work, we made regular random reliability checks to assess observer drift, and when we observed drift, we retrained. For the complete data set, 20% of interactions were coded by two observers who did not know which video segments served as reliability trials. Final Cohen’s kappa was .74 across Verbalizations, .76 for Verbal Reactive Aggression, .77 for Verbal Proactive Aggression, and .81 for Verbal Other. Variables termed Verbal Reactive Aggression and Verbal Proactive Aggression were created for each child by summing the number of statements of that type that they made over the course of the first or second game, respectively. See Supplemental Materials C for initial validation findings on the video game procedure.

### Statistical approach for primary analyses

To assess whether children’s emotion related to their aggression, we created multi-level models in which within-person emotion variables (ten 30-second intervals per task) related to between-person aggression variables. We ran 20 models in total, with each model including one emotion variable (Happiness, Sadness, Anger, Anxiety, Neutral) in one task (Search, Planning), along with either the Behavioral or Verbal Aggression variables. Of note, both Behavioral Proactive Aggression and Behavioral Reactive Aggression were included in the same models, to account for their correlation, and the same approach was taken for analyses involving Verbal Aggression. Because of this approach to the covariance between the subtypes of aggression, aggression variables were treated as predictors, and emotion variables were treated as outcomes. However, our goal was simply to examine relations between the two sets of variables. We made no assumptions regarding direction of effects, particularly given that data collection for the emotion variables preceded data collection for the aggression variables.

Following Bolger and colleagues (2003), analyses were multi-level to allow for more precise measurement of emotion, since we assessed emotion on a second-by-second basis and aggregated these assessments into ten 30-second intervals per task. Moreover, analyses followed the recommendations of Krull and MacKinnon (1999) to apply MacKinnon and Dwyer’s (1993) approach to multi-level models. Because many emotion and aggression variables were skewed, and to handle missing data, we used full information maximum likelihood with robust standard errors in primary analyses. Given that we ran 20 models, we assessed significance with and without a Bonferroni correction of *p* < .0025. Throughout multi-level modeling, we used Bayesian estimation (which treats parameters as random variables with probability distributions) to make the analyses less computationally demanding, given the large number of parameters involved (Muthén, 2010).

#### Analytic approach to round-robin design

When researchers observe peer interaction, they typically do not consider the impact of the particular peers with whom children interact. For example, if we observed children’s tendency to express anger toward their peers on the playground, this approach would ignore the fact that some peers may elicit more anger than others. Thus, in this example, children’s anger scores would be confounded with the peers with whom they happened to interact, a phenomenon referred to as a “partner effect” (Kenny et al., 2006). In the current study, we solved this problem through the use of a round-robin design in which children’s emotion was assessed separately as they interacted with each other child in their (typically four-person) group, a design approach suggested by Kenny and colleagues.

However, our use of a round-robin design to address the issue of partner effects created interdependence in the data set. Namely, we could not assume independence of each child’s emotion across partners or independence of each partner’s influence on the children with whom he/she interacted. We addressed this interdependence through a modeling approach uniquely suited to this research design suggested by Kenny (personal communication, 2018), which we have used in two other published papers to date (Hubbard et al., 2022, 2023).

Specifically, we made four modeling decisions which in combination address the interdependence created by the round-robin nature of the data. In particular, we further built up the model to: (a) include all six dyads per group in wide format, (b) constrain intercepts to be equal and variances to be equal for all 12 emotion variables (each of four children’s emotion with each of three partners per group), (c) set covariances among all 12 emotion variables to 0, and (d) set the 12 relations between proactive aggression and emotion variables to be equal across dyads and children within each group, as well as the 12 relations between reactive aggression and emotion variables. In combination, the constraints imposed meant that the data set was represented by two levels, with Level 1 representing the 10 assessments per emotion task and Level 2 representing the 52 groups of (typically four) children. Levels were not included for dyad or child, because values at these levels were constrained to be equal. See Supplemental Materials D for an illustration of the data sets and the nesting structure.

Admittedly, these constraints removed between-child and between-dyad variance, in that they implied invariant effects across children and dyads. However, the constraints were needed to address the interdependence in the data created by the round-robin design, an issue which could not be addressed via alternative methods (Kenny, personal communication, 2018). By applying the constraints, analyses provided one estimate across all 12 child-partner combinations, which allowed us to address the question of whether children’s emotion across partners in the dyadic interaction procedures predicted their reactive or proactive aggression in the video game procedure.

## Results

### Data aggregation, descriptive statistics, and correlations

Descriptive statistics and task differences for emotion variables are displayed in Table 1. For these purposes only, we further aggregated emotion variables across all ten intervals of each task. As can be seen in Table 1, many children retained a neutral expression over the course of one interval of one task with one partner. However, only two children remained neutral in their emotion expression throughout all three search tasks, and no child remained neutral throughout all three planning tasks. For a full accounting of the multi-level relations amongst emotion variables, see (Hubbard et al., 2023). Descriptive statistics are provided for aggression variables in Table 2, along with correlations amongst these variables.

**Table 1.** Descriptive statistics and task differences for emotion variables

	Search tasks					Planning tasks					
	Min	Max	*M*	*SD*	Skew	Min	Max	*M*	*SD*	Skew	Task Difference (*F)*
Happiness	0.00	100.00	8.20	15.43	2.98	0.00	100.00	14.24	19.67	1.91	97.12***
Sadness	0.00	35.03	0.13	1.30	13.83	0.00	81.88	0.08	1.35	40.48	2.81
Anger	0.00	76.62	1.32	4.77	6.48	0.00	58.30	0.47	2.45	10.20	55.40***
Anxiety	0.00	100.00	1.02	5.19	9.21	0.00	100.00	0.83	4.99	10.71	1.77
Neutral emotion	0.00	100.00	89.11	17.56	−2.46	0.00	100.00	83.85	21.11	−1.81	59.50***

*Note. N* = 6,060 (202 children × 3 partners × 10 intervals); Min = minimum; Max = maximum; *M* = mean; *SD* = standard deviation; *** *p* < .001.

**Table 2.** Descriptive statistics and bivariate correlations for aggression variables

						Bivariate Correlations			
	Min	Max	*M*	*SD*	Skew	1	2	3	4
1. Behavioral proactive aggression	0.00	297.89	55.26	75.64	1.65	—	—	—	—
2. Behavioral reactive aggression	0.00	298.46	70.03	83.96	1.39	.66***	—	—	—
3. Verbal proactive aggression	0.00	13.00	.92	2.12	3.30	.31***	.26***	—	—
4. Verbal reactive aggression	0.00	22.00	1.94	2.98	2.92	.19*	.32***	.36***	—

*Note. N* = 158 children; Min = minimum; Max = maximum; *M* = mean; *SD* = standard deviation; *p < .05; ***p < .001.

### Primary analyses

For each of the 20 models, we assessed model fit using Posterior Predictive Checks (PPCs), a fit statistic appropriate for Bayesian models. All 20 PPCs indicated good model fit, which is suggested when the values fall between .50 and .95 (Kruschke, 2021) For all 20 models, the PPPSs ranged from .52 to .85, suggesting that the data fell within the 95% confidence interval of simulated data.

We hypothesized that both Behavioral and Verbal Reactive Aggression would relate positively to Happiness, Sadness, Anger, and Anxiety, but negatively to Neutral Emotion. This prediction was supported for seven of 20 relations. Behavioral Reactive Aggression in the video games was positively related to Anger in the search tasks, and Verbal Reactive Aggression in the video games was positively related to Happiness, Anger, and Anxiety in the search tasks. Furthermore, verbal Reactive Aggression in the video games was positively related to Happiness in the planning tasks. Finally, verbal Reactive Aggression in the video games was negatively related to Neutral Emotion in both the search and planning tasks (see Table 3).

**Table 3.** Multi-level models of emotion and reactive versus proactive aggression

	Search tasks				Planning tasks			
	Reactive aggression		Proactive aggression		Reactive aggression		Proactive aggression	
	Est	PSD	Est	PSD	Est	PSD	Est	PSD
Behavioral aggression								
Happiness	.006	.007	−.028***	.008	.002	.009	−.018*	.001
Sadness	.000	.000	−.001*	.000	.000	.000	.000	.000
Anger	.002*	.002	−.001	.002	.000	.000	.000	.001
Anxiety	−.003	.002	.000	.002	.001	.001	−.001	.001
Neutral emotion	−.005	.008	.034***	.009	.004	.010	.021*	.012
Verbal aggression								
Happiness	.438***	.162	−.549*	.255	.970***	.210	−.654*	.326
Sadness	.006	.006	−.004	.009	.003	.001	.001	.002
Anger	.147***	.060	−.036	.038	−.010	.012	.006	.018
Anxiety	.084**	.040	−.076	.062	−.018	.027	.001	.042
Neutral emotion	−.573***	.186	.616**	.292	−.934***	.227	.744**	.353

*Note.* Estimates are unstandardized. Est = Estimate; PSD = Posterior Standard Deviation; *p< .05; **p< .01; ***p< .001.

Estimates which are significant at the *p* < .001 level remain significant with Bonferroni corrections for Type 1 error, whereas estimates which are significant at the *p* < .05 or .01 levels do not.

We hypothesized that both Behavioral and Verbal Proactive Aggression would relate negatively to Happiness, Sadness, Anger, and Anxiety, but positively to Neutral Emotion. This prediction was supported for nine of 20 relations. Both Behavioral and Verbal Proactive Aggression in the video games were negatively related to Happiness in both the search and planning tasks. In addition, a significant negative relation also emerged between Behavioral Proactive Aggression in the video games and Sadness in the search tasks. Finally, both Behavioral and Verbal Proactive Aggression in the video games positively related to Neutral Emotion in both the search and planning tasks. With the Bonferroni correction of *p* < .0025, seven of the 16 significant effects described above maintained significance (see the Note under Table 3). The distinction between effects which did and did not maintain significance using this correction is addressed throughout the discussion below.

## Discussion

The first goal of the current study was to investigate connections between children’s emotion and the functions of aggression using observational or behavioral assessments of both constructs. Given the theoretical importance of this link, we aimed to expand our understanding of the connection using state-like or situational measures of both constructs, as opposed to the more common approach of assessing emotion or aggression through questionnaire methods, in which individuals accumulate perceptions of their own or others’ emotion or aggression across situations to develop and report on more stable traits in terms of emotion or aggression. The second goal was to advance our understanding of the range of emotions associated with reactive aggression by observing children’s happiness, sadness, anger, and anxiety. The final goal was to include observation of neutral emotion to address the question of whether proactive aggression is unrelated or negatively related to emotion.

### Reactive aggression and a range of heightened emotions

We hypothesized that reactive aggression would be positively related to happiness, sadness, anger, and anxiety, but negatively related to neutral emotion. Of the 20 relations between either behavioral or verbal reactive aggression and each of the five emotions variables in one of the two tasks, seven associations supported this prediction (five with Bonferroni correction), consolidating support for the emotional nature of reactive aggression. To our knowledge, this is only the second study to take a discrete emotions approach (Izard, 1993) to the study of reactive aggression. We expanded on the work of Moore and colleagues (2019), who explored links between reactive aggression and discrete emotions in daily diaries, by examining these links using observations of emotion.

Anger is the emotion most closely tied to reactive aggression both theoretically (Berkowitz, 1993) and empirically (e.g., Hubbard et al., 2002; Ostrov et al., 2013). In the current study, positive relations emerged between anger and both behavioral and verbal reactive aggression in the search tasks. Those children who expressed more anger as they searched for missing objects with peers also were more likely to prevent their opponent from earning points and make aggressive remarks suggesting retaliation during the video game. This linking of observational/behavioral measures of anger and reactive aggression across contexts reinforces prior theory and evidence.

Extant work suggests that reactive aggression is associated not only with anger, but also anxious symptoms (e.g., Fite et al., 2014; Fung et al., 2015) and increased variability in daily fear (Moore et al., 2019). In the current study, children who expressed more anxiety during the search tasks made more verbal remarks that were reactively aggressive during the video game. These findings support Berkowitz and Harmon-Jones’ (2004) theorizing around reactive aggression and heightened negative emotion beyond anger. Of course, in the moment that youth reactively aggress, they likely experience anger more than other negative emotions. However, these results suggest that children who reactively aggress may experience broad-based negative emotion more often or more intensely than their peers, even when they are not aggressive.

Beyond anger and anxiety, we predicted that reactive aggression would be positively related to sadness; however, these associations did not emerge. These null results stand in contrast to prior literature highlighting connections between reactive aggression and both depressive symptoms (e.g., Preddy et al., 2014; Yang et al., 2024) and suicidality (Hartley et al., 2018). However, both tasks elicited less sadness than other emotions (see Table 1). In future work, researchers should use tasks specifically designed to elicit sadness and relate observations of sadness to behavioral or observational measures of youth reactive aggression.

The daily diary study by Moore and colleagues (2019) raised the intriguing possibility that reactive aggression may have ties not only to negative emotions, but also to positive emotions. Although reactive aggression was related to lower daily levels of adolescents’ happiness in that study, it was also associated with greater responses of happiness when positive events occurred. This result is mirrored in the current finding of a positive link between verbal reactive aggression and children’s happiness in both tasks. Of course, in our observational study, it was not possible to untangle level of happiness from happy responses to specific positive events. Even so, the more children expressed happiness during the dyadic interactions, the more they made retaliatory aggressive statements during the video games. This finding implies that children who display reactive aggression may be particularly emotionally labile with respect to both positive and negative emotions (Slaughter et al., 2020), although this interpretation would be enhanced by the study of positive emotions other than happiness.

Beyond connections between reactive aggression and anger, anxiety, and happiness, a negative relation emerged between verbal reactive aggression and neutral emotion in both tasks. Of course, to some extent, these findings are complementary; the more time children spend displaying emotions such as happiness or anger, the less time remains to express neutral affect. Nonetheless, the children who struggled the most to remain calm and composed during the dyadic tasks were the most likely to display reactive aggression during the video games. This pattern highlights the challenges that reactively aggressive children face in regulating emotion (e.g., Calvete & Orue, 2012; Marsee & Frick, 2007; Ostrov et al., 2013), likely because of both decreased parasympathetic functioning and hyperarousal of the sympathetic nervous system (Hubbard et al., 2002; Moore et al., 2018; Scarpa et al., 2010; Xu et al., 2014).

Two patterns in these findings bear explanation. First, the positive associations between reactive aggression and both anger and anxiety emerged only in the context of the search tasks. These tasks were by design more frustrating than the planning tasks and therefore elicited higher levels of and more variability in negative emotion (see Table 1), offering a possible explanation for this pattern. Second, the connections between reactive aggression and anxiety, happiness, and neutral emotion emerged for verbal aggression but not for behavioral aggression, suggesting that our verbal measure of reactive aggression may have been more sensitive than our behavioral measure. Children may have been more hesitant to make retaliatory remarks to a strange peer than to “zap” that peer in the context of a video game, a setting in which many children may consider that behavior to be expected.

### Proactive aggression, neutral emotion, and lack of emotion

We hypothesized that proactive aggression would be negatively related to happiness, sadness, anger, and anxiety, but positively related to neutral emotion. Of 20 relations between proactive aggression and emotion, nine associations supported this prediction (two with Bonferroni correction), bolstering support for the unemotional nature of proactive aggression through observational methodology. Of note, the findings for proactive aggression were less robust to Bonferroni correction than the results for reactive aggression, although they were also more consistent across models.

In fact, proactive aggression was positively related to neutral emotion across both behavioral and verbal measures of aggression and across both tasks, suggesting a robust association. Children who maintained a more neutral emotional expression during the dyadic tasks were more likely to “zap” their opponent to win the video game, while at the same time making remarks suggesting willingness to aggress to win the game. These findings are consistent with previous null relations between proactive aggression and anger (e.g., Calvete & Orue, 2012; Hubbard et al., 2002; Jambon et al., 2019; Song et al., 2020; Xu et al., 2009), depressive/anxious symptoms (Fite et al., 2014), and negative emotionality or negative emotional lability (Slaughter et al., 2020; Vitaro et al., 2002). Given that the search tasks in particular were quite frustrating, the ability to remain emotionally neutral implies considerable calm and composure on the part of these children, perhaps due to strong emotion regulation skills (e.g., Ostrov et al., 2013; Rathert et al., 2011), well-developed parasympathetic regulation (Moore et al., 2018), and low baseline sympathetic arousal (e.g., Raine et al., 2014; Scarpa et al., 2010).

Furthermore, proactive aggression was negatively linked to happiness across both measures of aggression and both tasks as well, again indicating an especially consistent finding. The less children expressed happiness during the dyadic tasks, the more they displayed both behavioral and verbal proactive aggression during the video games. This finding parallels the sole significant result to emerge for proactive aggression from the daily diary study by Moore and colleagues (2019); in that investigation, proactive aggression predicted less variability in adolescents’ daily happiness. To our knowledge, these are the only two studies to take a discrete emotions approach to exploring the unemotional nature of proactive aggression, and interestingly, they converge on happiness as a key emotion. This focus on lack of happiness suggests that youth may seek to increase the sensation of positive emotion by aggressing for reward (Portnoy et al., 2014; Raine, 2002; Sijtsema et al., 2010; Xu et al., 2014).

However, we did not find negative relations between proactive aggression and anger or sadness, in contrast to previous work (Ostrov et al., 2013; Preddy et al., 2014). Interestingly, both of these studies relied on teacher report of emotion, as opposed to our use of observational methods. This methodological difference may be at the root of the differential findings, as may the contextual differences of school-versus laboratory-based assessment.

One additional finding emerged, a negative relation between sadness and behavioral proactive aggression in the search task. However, although this finding was statistically significant, the effect size was quite small. For this reason, we hesitate to interpret it further.

Importantly, both the current study and previous studies typically assess proactive aggression and emotion in different contexts, preventing firm conclusions about youths’ emotion as they actually engage in proactive aggression. To our knowledge, only one investigation assessed emotion at the moment that proactive aggression occurs, and this investigation took a physiological approach (Moore et al., 2018). Findings suggested that, at the moment that children engage in proactive aggression, they experience both low levels of sympathetic arousal and high levels of parasympathetic functioning. This study, in our view, provides the most direct window into the emotional state of youth during proactively aggressive acts.

### Limitations and future directions

Like all investigations, the current study is characterized by limitations which provide suggestions for future researchers. First, this study marks the first use of the video game procedure to assess reactive and proactive aggression, a procedure that has not been validated through associations with other measures of the functions of aggression. Of course, to some extent, this investigation itself provides validation, given the strong parallels between our findings and prior work. However, future researchers should focus on developing and validating robust behavioral/observational measures of reactive and proactive aggression.

Second, the video game procedure assessed physical (pedal presses) and verbal (aggressive statements) forms of aggression, as opposed to relational forms. The distinction between forms and functions of aggression is an important one (e.g., Little et al., 2003), and the overlap between the two constructs is complex (e.g., Marsee et al., 2011; Murray-Close & Ostrov, 2009), suggesting the need for future researchers to expand our work using a procedure that captures relational reactive and relational proactive aggression.

Third, although we did not emphasize the timeline throughout the paper, we assessed emotion when children were nine years old and then the functions of aggression one year later when they were ten years old. Given the lack of measurement of both constructs at both time points, as well as equivocal findings on the directionality of the developmental link between emotion and aggression (e.g., McLaughlin et al., 2011; Ostrov et al., 2013), we chose not to focus on issues of temporal sequence or prediction. However, it is noteworthy that the findings reported here linked emotion and aggression over the span of one year. Even so, future researchers of longitudinal links between emotion and aggression should assess both constructs at each time point to untangle their directionality.

Fourth, the sample was high-risk, in that many children came from low-income families and/or had been referred by CPS to the larger project. These characteristics may be a strength, in that they increase the diversity of samples in studies of the functions of aggression and emotion. However, they also are a limitation, in that our participants may have been especially motivated by the prizes and monetary rewards offered. For this reason, future researchers should replicate our findings with a more normative sample.

### Conclusions

In conclusion, the current study took an observational/behavioral approach to the study of emotion and the functions of aggression in children. Findings largely supported and increased confidence in prior work suggesting that reactive aggression is emotional in nature, whereas proactive aggression is unemotional. Furthermore, results highlighted the roles of the discrete emotions of anger, anxiety, and happiness in reactive aggression and suggested that proactive aggression may be characterized by neutral emotional displays, as well as a lack of happiness.

## Supporting information

10.1017/S0954579426101394.sm001 Hubbard et al. supplementary material 1 Hubbard et al. supplementary material

10.1017/S0954579426101394.sm002 Hubbard et al. supplementary material 2 Hubbard et al. supplementary material

10.1017/S0954579426101394.sm003 Hubbard et al. supplementary material 3 Hubbard et al. supplementary material

10.1017/S0954579426101394.sm004 Hubbard et al. supplementary material 4 Hubbard et al. supplementary material

## Data Availability

All data sets and analyses are available at https://osf.io/ejdms/?view_only=724b3f89a425413c86443760ead74722, and we follow JARS (Kazak, 2018). Data aggregation and descriptive statistics were conducted using the Statistics Package for the Social Sciences (v. 28). Primary analyses were conducted using *M*plus (v. 8.7; Muthén & Muthén, 1998-2017).

## Supplementary material

The supplementary material for this article can be found at https://doi.org/10.1017/S0954579426101394.

## Pre-registration

This study was not pre-registered, because data were collected at a time when pre-registration was not common.

## Clinical trials.gov

Intervening Early with Neglected Children; https://clinicaltrials.gov/show/NCT02093052.
