# Supplementary material for: Links between observational measures of children’s emotion and reactive versus proactive aggression
Source: Dev Psychopathol. 2026 Mar 30:1–11. Online ahead of print. doi: 10.1017/S0954579426101394 (PMC13107196; doi:10.1017/S0954579426101394)
Supplement: Hubbard et al. supplementary material 1 — Hubbard et al. supplementary material [file S0954579426101394sup001.docx]

**Supplemental Materials A**

**CPS-Status Differences in Study Variables**

We compared CPS-referred and non-CPS-referred children on study variables. No differences emerged (see Table 1 below).

**Table 1**

*Differences on Study Variables Between CPS-Referred and Non-CPS-Referred Children*

|  | *M*_CPS_ | *M*_Non-CPS_ | *F* | *p* | *η^2^* |
| --- | --- | --- | --- | --- | --- |
| Emotion: |  |  |  |  |  |
| Search Tasks: |  |  |  |  |  |
| Happy | 8.64 | 7.60 | .91 | .34 | .01 |
| Sad | .17 | .10 | 2.63 | .11 | .01 |
| Angry | 1.55 | 1.12 | 2.76 | .10 | .01 |
| Anxious | 1.25 | .80 | 2.92 | .14 | .01 |
| Neutral | 88.15 | 90.18 | 2.47 | .12 | .01 |
| Planning Tasks: |  |  |  |  |  |
| Happy | 13.72 | 14.94 | .61 | .44 | .00 |
| Sad | .07 | .09 | .15 | .70 | .00 |
| Angry | .59 | .32 | 2.11 | .13 | .02 |
| Anxious | 1.04 | .62 | 3.10 | .13 | .02 |
| Neutral | 84.35 | 83.21 | .50 | .48 | .00 |
| Aggression: |  |  |  |  |  |
| Behavioral Proactive Aggression | 57.56 | 51.76 | .22 | .64 | .00 |
| Behavioral Reactive Aggression | 69.81 | 70.36 | .00 | .97 | .00 |
| Verbal Proactive Aggression | .94 | .90 | .01 | .93 | .00 |
| Verbal Reactive Aggression | 1.86 | 2.05 | .15 | .70 | .00 |

*Note: η^2^* = partial eta squared
