# Supplementary material for: Links between observational measures of children’s emotion and reactive versus proactive aggression
Source: Dev Psychopathol. 2026 Mar 30:1–11. Online ahead of print. doi: 10.1017/S0954579426101394 (PMC13107196; doi:10.1017/S0954579426101394)
Supplement: Hubbard et al. supplementary material 2 — Hubbard et al. supplementary material [file S0954579426101394sup002.docx]

**Supplemental Materials B**

**Intervention Group Differences on Study Variables**

We compared children who received Intervention ABC to those who received Intervention DEF on study variables. No differences emerged (see Table 1 below).

**Table 1**

*Differences on Study Variables Between Children Who Received Intervention ABC and Intervention DEF*

|  | *M_ABC_* | *M_DEF_* | *F* | *p* | *η^2^* |
| --- | --- | --- | --- | --- | --- |
| Emotion: |  |  |  |  |  |
| Search Tasks: |  |  |  |  |  |
| Happy | 7.98 | 9.21 | .54 | .47 | .01 |
| Sad | .11 | .23 | 1.90 | .17 | .02 |
| Angry | 1.57 | 1.53 | .01 | .92 | .00 |
| Anxious | 1.18 | 1.31 | .08 | .77 | .00 |
| Neutral | 89.09 | 87.34 | .71 | .40 | .01 |
| Planning Tasks: |  |  |  |  |  |
| Happy | 13.91 | 13.56 | .03 | .86 | .00 |
| Sad | .08 | .07 | .01 | .93 | .00 |
| Angry | .58 | .60 | .02 | .89 | .00 |
| Anxious | 1.01 | 1.07 | .02 | .88 | .00 |
| Neutral | 84.23 | 84.46 | .01 | .91 | .00 |
| Aggression: |  |  |  |  |  |
| Behavioral Proactive Aggression | 63.17 | 52.63 | .47 | .50 | .01 |
| Behavioral Reactive Aggression | 72.13 | 67.77 | .06 | .80 | .00 |
| Verbal Proactive Aggression | 1.32 | .60 | 2.82 | .10 | .03 |
| Verbal Reactive Aggression | 2.36 | 1.42 | 1.93 | .17 | .02 |

*Note: η^2^* = partial eta squared
