# Supplementary material for: Links between observational measures of children’s emotion and reactive versus proactive aggression
Source: Dev Psychopathol. 2026 Mar 30:1–11. Online ahead of print. doi: 10.1017/S0954579426101394 (PMC13107196; doi:10.1017/S0954579426101394)
Supplement: Hubbard et al. supplementary material 3 — Hubbard et al. supplementary material [file S0954579426101394sup003.docx]

**Supplemental Materials C**

**Initial Validation Findings on Video Game Procedure**

We evaluated relations between children’s four aggression scores [Behavioral (Verbal) Reactive (Proactive) Aggression] and their self-reported emotion regulation, emotional lability, symptoms of Attention Deficit Hyperactivity Disorder (ADHD), and symptoms of various anxiety disorders. Data on emotion regulation and emotional lability were collected using the Emotion Regulation Checklist (ERC; Shields & Cicchetti, 1997), and data on ADHD and anxiety symptoms were collected using the Children’s Interview for Psychiatric Syndromes (ChIPS; Rooney et al., 1999; number of symptoms reported). Based on research reviewed in this paper, we hypothesized that emotional lability and anxiety symptoms would be positively related to reactive aggression and negatively related or unrelated to proactive aggression. In contrast, we predicted that emotion regulation would be positively related to proactive aggression but negatively related to reactive aggression. Based on extant research, we hypothesized that ADHD symptoms would be positively related to reactive aggression but negatively related to proactive aggression (Card & Little, 2006; Conner; Dodge et al., 1997; Vitaro et al., 2002). Findings are provided in Table 1.

These results provide support for the criterion validity of the video game procedure, and the primary findings of the paper also provide some initial support for its construct validity. Of course, confidence in the validity of the procedure will increase when additional research relates it to other established measures of reactive and proactive aggression, an important step for future scholars.

**Table 1**

*Correlations Between Aggression Scores and Emotion Regulation, Emotional Lability, and Symptoms of Attention Deficit Hyperactivity Disorder and Anxiety Disorders*

|  | Bivariate Correlations | | | | Partial Correlations | | | |
| --- | --- | --- | --- | --- | --- | --- | --- | --- |
|  | BPA | BRA | VPA | VRA | BPA | BRA | VPA | VRA |
| Emotion Regulation | .08 | -.05 | .14* | -.01 | .06 | .00 | .15* | -.07 |
| Emotional Lability | -.18* | .17* | -.05 | .01 | -.09* | .08 | -.06 | .11 |
| ADHD | -.20* | .22* | -.07 | .06 | -.14* | .18* | -.10 | .10 |
| Social Phobia | -.14* | .21** | -.08 | .06 | -.03 | .04 | -.04 | .05 |
| Separation Anxiety | -.11 | .14* | -.06 | .09 | .00 | .10* | -.05 | .08 |
| GAD | -.16* | .20** | -.27* | .26* | -.11* | .12* | -.14* | .12* |

*Note:* BPA = Behavioral Proactive Aggression; BRA = Behavioral Reactive Aggression; VPA = Verbal Proactive Aggression; VRA = Verbal Reactive Aggression; ADHD = Attention Deficit Hyperactivity Disorder; GAD = Generalized Anxiety Disorder; partial correlations control for the opposite function of aggression within behavioral or verbal aggression (e.g., correlations for BPA control for BRA and correlations for VRA control for VPA); * *p <* .05; ** *p* < .01; *** *p* < .001

**References**

Card, N.A., & Little, T.D. (2006). Proactive and reactive aggression in childhood and adolescence: A meta-analysis of differential relations with psychosocial adjustment. *International Journal of Behavioural Development, 30,* 466-480. doi: [10.1177/0165025406071904](https://psycnet.apa.org/doi/10.1177/0165025406071904)

Connor, D.F., Chartier, K.G., Preen, E.C., & Kaplan, R.F. (2010). Impulsive aggression in attention-deficit/hyperactivity disorder: Symptom severity, co-morbidity, and attention-deficit/hyperactivity disorder sybtype. *Journal of Child and Adolescent Psychopharmacology, 20,* 119-126. doi:[10.1089/cap.2009.0076](https://doi.org/10.1089/cap.2009.0076)

Dodge, K.A., Lochman, J.E., Harnish, J.D., Bates, J.E., & Pettit, G.S. (1997). Reactive and proactive aggression in school children and psychiatrically impaired chronically assaultive youth. *Journal of Abnormal Psychology, 106,* 37-51. doi:[10.1037//0021-843x.106.1.37](https://doi.org/10.1037/0021-843x.106.1.37)

Shields, A., & Cicchetti, D. (1997). Emotion regulation among school-age children: The development and validation of a new criterion Q-sort scale. *Developmental Psychology, 33,* 906-916. doi: 10.1037/0012-1649.33.6.906

Rooney, M.T., Fristad, M.A., Weller, E.B., & Weller, R.A. (1999). *Administration manual for the ChIPS*. American Psychiatric Association.

Vitaro, F., Brendgen, M., & Tremblay, R.E. (2002) Reactively and proactively aggressive children: Antecedent and subsequent characteristics. *Journal of Child Psychology and Psychiatry and Allied Disciplines, 43,* 495-505. doi:[10.1111/1469-7610.00040](https://doi.org/10.1111/1469-7610.00040)
