# Supplementary material for: Links between observational measures of children’s emotion and reactive versus proactive aggression
Source: Dev Psychopathol. 2026 Mar 30:1–11. Online ahead of print. doi: 10.1017/S0954579426101394 (PMC13107196; doi:10.1017/S0954579426101394)
Supplement: Hubbard et al. supplementary material 4 — Hubbard et al. supplementary material [file S0954579426101394sup004.docx]

**Supplemental Materials D**

**Illustration of the Multiple Levels of the Data Set**

**
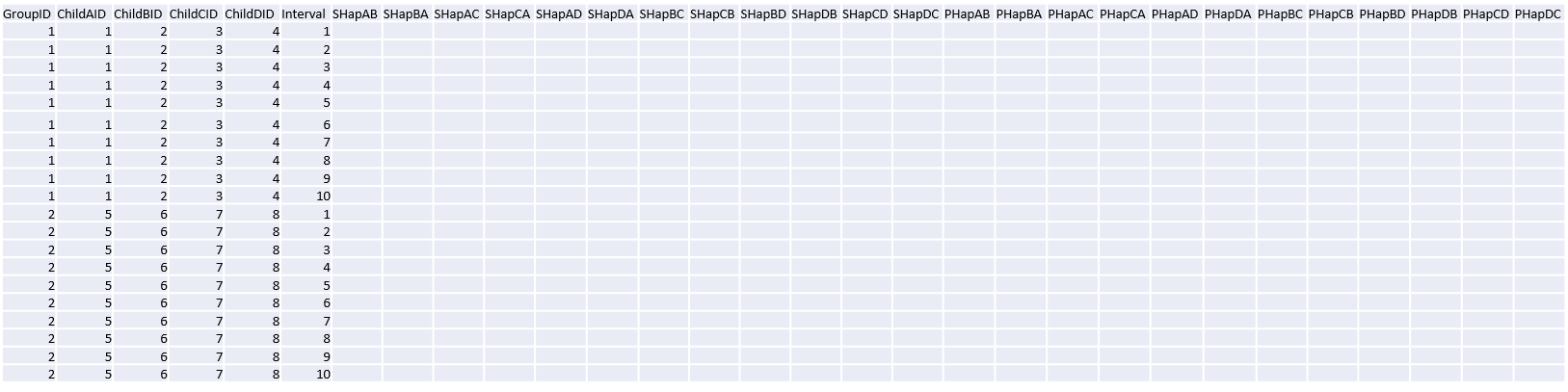
Illustration of the Emotion Data Set**

Repeat for remaining 4 emotions

Repeat for

remaining 4

emotions

GroupID = ID number of each of the 52 groups who completed the emotion task

ChildAID, ChildBID, ChildCID, ChildDID = ID number of each of the four children in the group

Interval = the ten 30-second intervals of the 5-minute task

Emotion variable names:

- 1^st^ letter: S or P = Search or Planning Task
- 2^nd^-4^th^ letters: Hap = Happy, Sad = Sad, Ang = Angry, Anx = Anxious, Neu = Neutral
- 5^th^-6^th^ letters: AB, BA, AC, CA, AD, DA, BC, CB, BD, DB, CD, DC = 1^st^ letter represents the child expressing emotion; 2^nd^ letter represents dyadic partner

Thus, the first unfilled cell represents the percentage of the first 30-second interval of the search task that Child A expressed happiness when with Child B.

Repeat for ChildIDs 9-202

Repeat for GroupIDs 3-52

**Illustration of the Aggression Data Set**

| ChildID | BhRcAg | BhPrAg | VbRcAg | VbPrAg |
| --- | --- | --- | --- | --- |
| 1  Repeat for ChildIDs 2-158 |  |  |  |  |

ChildID = ID number of each of the 158 children who completed the aggression task

Aggression variable names:

- 1^st^-2^nd^ letters: Bh or Vb = Behavioral or Verbal
- 3^rd^-4^th^ letters: Rc or Pr = Reactive or Proactive
- 5^th^-6^th^ letters: Ag = Aggression

Thus, the first unfilled cell represents the behavioral reactive aggression score for ChildID 1.
